# Amplitude Control Method of Magnetic Flux Vertical Modulation Structure for TMR Magnetic Sensor Based on Harmonic Component of Modulated Signal

**DOI:** 10.3390/mi12060722

**Published:** 2021-06-19

**Authors:** Junsheng Zhang, Mengchun Pan, Qingfa Du, Jiafei Hu, Kun Sun, Yang Yu, Xinmiao Zhang, Huihui Luo

**Affiliations:** College of Intelligence Science and Technology, National University of Defense Technology, Guizhou 410073, China; zhangjunshengNUDT@126.com (J.Z.); pmc_nudt@vip.163.com (M.P.); garfield_nudt@163.com (J.H.); ytsunkun@126.com (K.S.); yuyang0316@hotmail.com (Y.Y.); zxinmiao2020@163.com (X.Z.); luohuihui1126@163.com (H.L.)

**Keywords:** TMR magnetoresistance sensor, magnetic flux modulation, resonance control, temperature drift compensation

## Abstract

Magnetic flux vertical modulation method based on piezoelectric resonance can reduce the 1/*f* noise of tunnel magnetoresistance (TMR) magnetic sensor and significantly improves the low-frequency magnetic field detectivity. However, the amplitude variation of the modulation structure will lead to the instability of the sensor output. In order to improve the amplitude stability of the modulation structure, an amplitude control method based on the amplitude ratio of the first and second harmonic components of the modulated signal was proposed. Compared with the piezoelectric or capacitive feedback method, this method does not require an independent amplitude conversion circuit, and has the advantages of simple structure, high control efficiency and strong anti-interference ability. The experimental results showed that the amplitude and temperature drift of the modulated structure was significantly suppressed, which is of great significance for enhancing the adaptability of the TMR magnetic sensor to the application environments.

## 1. Introduction

**Figure 1:**
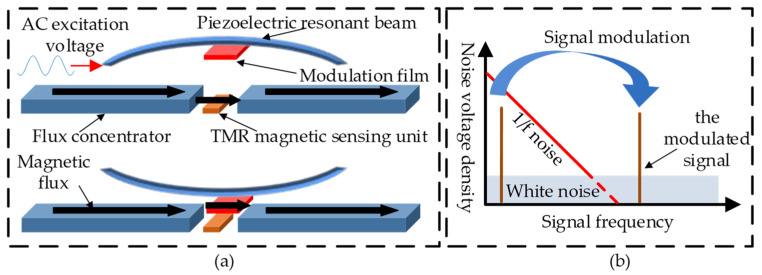
Magnetic flux vertical modulation based on piezoelectric resonance: (**a**) flux vertical modulation principle based on piezoelectric resonance; (**b**) noise suppression by modulation.

TMR magnetoresistance sensor has the advantages of high sensitivity, a large linear range and low power consumption. It has a great potential to be developed into a high-performance miniaturized magnetic sensor, and has a broad development prospect in the fields of geomagnetic navigation, field detection and nondestructive testing [1]. However, 1/*f* noise severely limits its low-frequency magnetic field detectivity [2,3]. In recent years, scholars and research teams from different countries have proposed a variety of flux modulation methods to suppress the 1/*f* noise of TMR magnetic sensor, but these methods generally have problems such as low modulation efficiency, leading to the deterioration of noise characteristics, and poor environmental adaptability [4,5,6,7,8,9]. The magnetic flux vertical modulation method based on piezoelectric resonance has a high modulation efficiency of 68.7% [10], which can reduce the 1/*f* noise of the TMR magnetic sensor by nearly two orders of magnitude, and greatly improve the low frequency magnetic field detectivity. The principle of magnetic flux vertical modulation based on piezoelectric resonance is shown as Figure 1. When Alternating current (AC) excitation is applied to the piezoelectric resonant beam, the modulation film with the effect of magnetic flux concentration vibrates periodically under the drive of the piezoelectric resonant beam. The TMR sensitive unit is set in the air gap of the flux concentrator. When the modulation film is far away from the air gap, the magnetic flux mainly passes through the TMR sensitive unit. When the modulation film is close to the air gap, it affects the flux concentration and results in most of the magnetic flux passing through the modulation film, as shown in Figure 1a. Therefore, with the periodic vibration of the modulation film, the magnetic field detected by the TMR sensitive unit will change from low frequency to high frequency. Since the 1/*f* noise decreases significantly with the increase of frequency, the modulated signal has a higher signal-to-noise ratio, as shown in Figure 1b.

**Figure 2:**
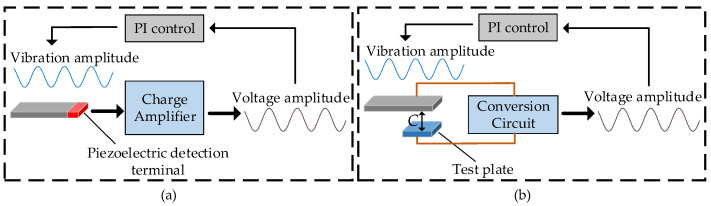
Piezoelectric and capacitive amplitude detection and control methods: (**a**) piezoelectric feedback amplitude control method; (**b**) capacitive feedback amplitude control method.

The mechanical and damping characteristics of the magnetic flux vertical modulation structure based on piezoelectric resonance are closely related to the environmental conditions, and the amplitude stability of the modulation structure will be affected by the external temperature change, or vibration shock. Common vibration monitoring and control methods of Micro–Electro–Mechanical Systems (MEMS) devices mainly include piezoelectric feedback amplitude control method and capacitive feedback amplitude control methods; these methods have been widely used in engineering practice [11,12,13]. The principle of piezoelectric feedback amplitude control method is to convert the change of amplitude into the change of the electrical signal at the detection terminal through the piezoelectric effect, as shown in Figure 2a. The principle of capacitive feedback amplitude control method is to convert amplitude change into detecting capacitance change, as shown in Figure 2b. These methods require independent amplitude conversion module and high precision detection circuit, which makes the control system more complex and the difficulty of process preparation increased obviously. Due to the pyroelectric phenomenon of piezoelectric materials and the temperature drift of the conversion circuit, the anti-interference ability and temperature drift suppression ability of these methods are insufficient.

We proposed an amplitude control method based on the harmonic component of the modulated signal. The innovation of this method is that the amplitude information of the structure is obtained directly by using the modulated output signal, and there is no need to build an independent conversion circuit on the existing sensor system. It has the characteristics of simple and efficient control loop, low process complexity, less interference coupling path and strong anti-interference ability. Moreover, this control method can effectively avoid the pyroelectric phenomenon of piezoelectric materials or the control failure caused by the temperature drift of the conversion circuit, and has a better adaptability to the variable temperature environment.

## 2. Principles and Methods

**Figure 3:**
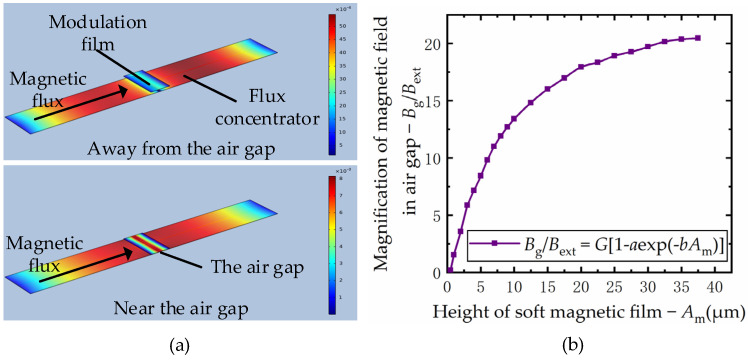
Finite element simulation of the relationship between the modulated signal *B*_g_ and the height of the modulation film *A*_m_: (**a**) simulation model of flux modulation structure; (**b**) simulation results of the relationship between magnetic field in air gap and the height of modulation film.

The air gap magnetic field under flux modulation (the modulated signal) is composed of alternating and static magnetic fields, and the alternating magnetic field has obvious harmonic component. The “magnetic field module” in COMSOL Multiphysics 5.4 (COMSOL Inc. Stockholm, Sweden) is used to build a finite element simulation model to analyze the nonlinear function relationship between the air gap magnetic field *B*_g_ and the height of the modulation film *A*_m_. The size of the flux concentrator was 1300 μm × 380 μm × 5 μm, the size of the modulation film was 200 μm × 390 μm × 5 μm and the relative permeability of the flux concentrator and the modulation film were set as 2000. The direction of the magnetic flux is shown as the arrow in Figure 3a. The influence of the height of the modulation film on the air gap magnetic field was analyzed, and the simulation results were shown as Figure 3b.

According to the results of finite element simulation model, the nonlinear relationship between the modulated signal *B*_g_ and the height of the modulation film *A*_m_ can be obtained by curve fitting method. Its mathematical description is as follows:

$$B_{g}{=GB}_{\mathrm{ext}}{[1-a\exp(-bA}_{m})]$$

where, *B*_ext_ is the size of the external magnetic field, *G* is the magnification of the concentrator and *GB*_ext_ is the air gap magnetic field intensity in the unmodulated state. *a* and *b* are modulation parameters, which are determined by the structural size of the modulation structure and the magnetic properties of the material. The modulation film vibrates periodically in the form of sine and cosine under the drive of piezoelectric structure, so the relationship among the modulated signal, the amplitude and frequency of the modulation film can be expressed as:

$$B_{g}{=GB}_{\mathrm{ext}}\left\{{1-a\exp(-\mathrm{bA}}_{0})\left[1+(-\mathrm{bA}\cos\left({2\pi f}_{m}t\right))+(-\mathrm{bA}\cos\left({2\pi f}_{m}t\right){)}^{2}/2+\ldots \right]\right\}$$

where, *A*_0_ is the initial height position of the modulation film, *A* is the amplitude of the modulation film and *f*_m_ is the vibration frequency of the modulated structure. The relation between the amplitude ratio of the first and second order harmonic components of the modulated signal and the amplitude of the modulation structure can be obtained as follows:

$${M=B}_{g(1)}{/B}_{g(2)}=2/(-\mathrm{bA})$$

**Figure 4:**
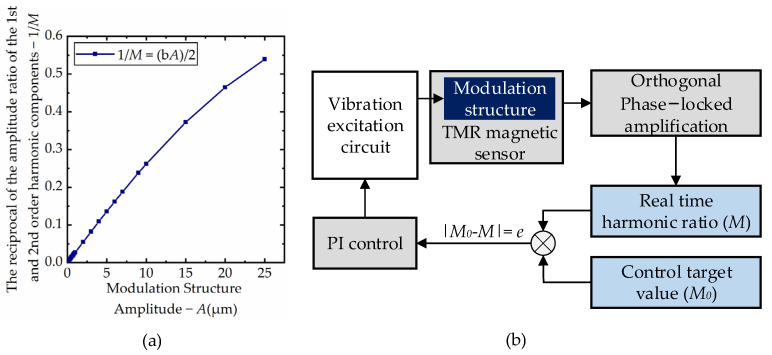
Modulated signal harmonic component and amplitude control method: (**a**) numerical simulation results of the relationship between the ratio of harmonic components and amplitude; (**b**) control schematic diagram.

A numerical simulation model was established to verify the relationship between the ratio of the first and second order harmonic components of the modulated signal and the amplitude. The magnetic field in the concentrator was set as 5000 nT, the modulation structure parameters *a* and *b* were set as 0.74 and 0.11, respectively, the vibration frequency of the modulation structure was set as 7800 Hz, and the initial amplitude *A*_0_ was set as 5 μm. When the amplitude of the modulation structure vibration was changed from 0.1 to 20 μm, the relation curve between the ratio of the first and second order harmonic components of the modulated signal and the amplitude is shown as Figure 4a. With the increase of the amplitude of the modulation film, the ratio of the first and second harmonic components of the modulated signal increased linearly, so it can be considered that it is feasible to use *M* to establish the control loop.

The amplitude control method based on the harmonic component of the modulated signal is shown in Figure 4b. Firstly, the amplitude of excitation voltage is adjusted to make the modulation structure reach the optimal vibration state. The high precision Analog Digital Converter (ADC) was used to collect the signal, and the amplitude of the first and second order harmonic components of the signal was extracted by the orthogonal phase-locked amplification method. The ratio of the two was the control target value *M*_0_, and the control threshold value *e*_n_ was set according to the vibration requirements. Then the same method was used to continuously collect and process the modulated signal during the operation of the magnetic sensor, and the real-time harmonic ratio *M* was obtained. Finally, the deviation *e* between *M* and the target value *M*_0_ was sent to the PI controller. By setting the parameters of the PI controller reasonably, the amplitude of the modulation structure can be quickly stabilized around the target value.

## 3. Experiment and Discussion

**Figure 5:**
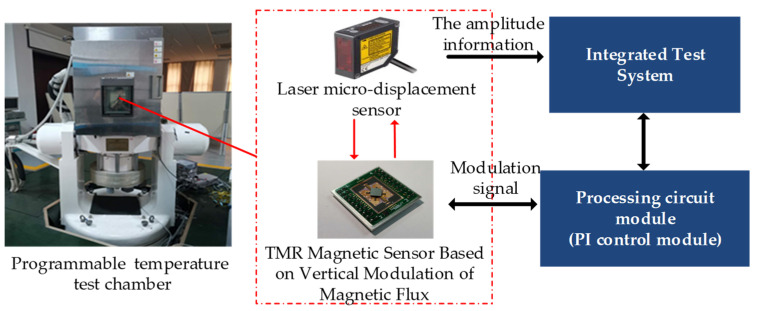
Schematic diagram of amplitude temperature drift compensation based on the harmonic component of the modulated signal.

In order to verify the effectiveness of the control method based on the ratio of the harmonic component of the modulated signal, a test system was built. The test system mainly includes: the TMR magnetic sensor, processing circuit module, programmable temperature test chamber (temperature fluctuation ≤ 0.5 °C), LK-HD500/LK-H008 (KEYENCE CORPORATION. Shanghai, China) type laser microdisplacement sensor (measurement range ±0.5 mm, repetition accuracy 0.005 μm, the operating temperature is 0–50 °C) and the integrated test platform based on the virtual instrument. The test system is shown as Figure 5. The TMR magnetic sensor and laser microdisplacement sensor were placed in the temperature test chamber. The amplitude of the modulation structure is tracked in real time by a microdisplacement sensor, and the amplitude information and the modulated signal are processed by an integrated testing system.

**Figure 6:**
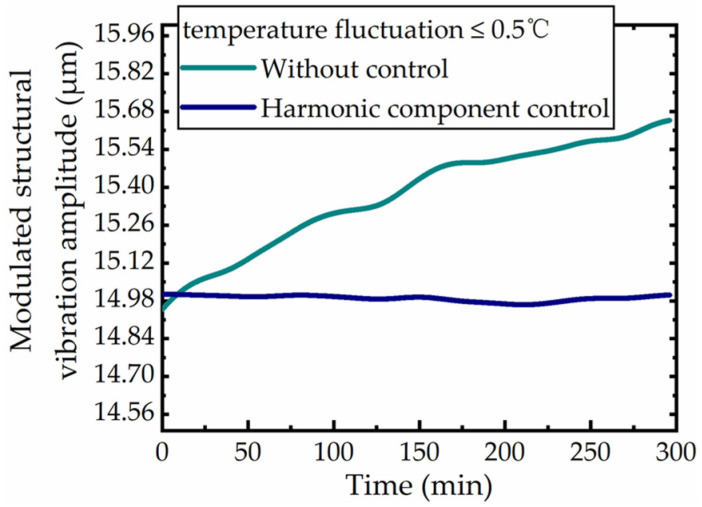
Control effect of the constant temperature condition.

First, the effectiveness of the amplitude control based on the harmonic component of the modulated signal was tested: the temperature test chamber was used to set the constant temperature environment to ensure the temperature fluctuation during the whole experiment was not more than 0.5 °C. The amplitude of the modulation structure was tracked for 300 min under controlled and uncontrolled conditions. The experimental results are shown as Figure 6. The experimental results show that the amplitude of the uncontrolled modulation structure increased continuously during the 5 h continuous sampling process, the amplitude shift reached 0.698 μm, and the change rate reached 4.7%. The amplitude has obvious jitter, which is mainly from the temperature fluctuation of the temperature chamber. This indicates that the modulation structure without control cannot maintain a stable state for a long time and its anti-interference ability is very limited. Under the same experimental conditions, the amplitude drift of the modulation structure was obviously reduced, the maximum fluctuation was less than 0.038 μm, the change rate was only 0.25%, the amplitude stability was improved about 18 times and the amplitude jitter phenomenon basically disappeared. The results proves that the amplitude control method based on harmonic component was effective under constant temperature condition, but the advantages of this method still need to be further discussed.

**Figure 7:**
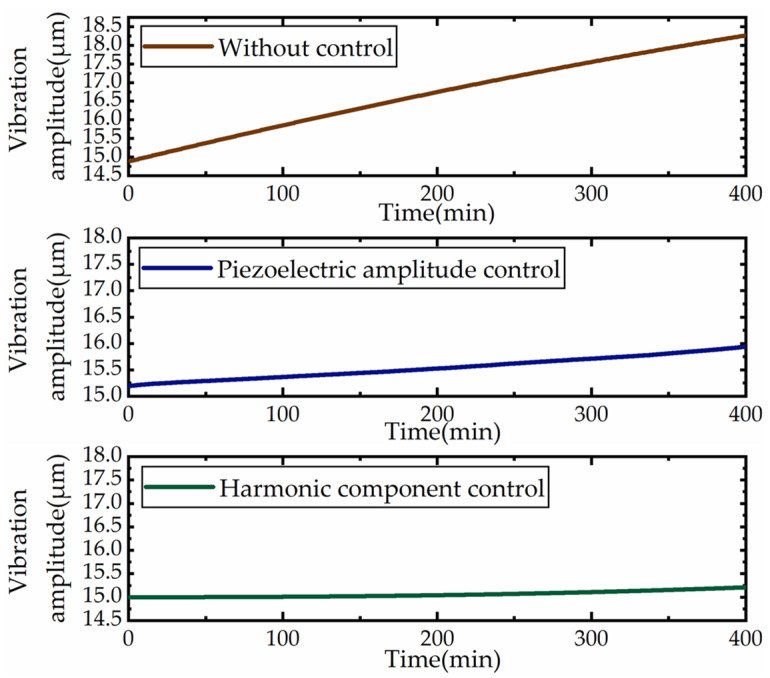
Control effect of the variable temperature condition.

Then the effect of amplitude control under variable temperature was tested by changing the experimental environment. The programmable temperature test chamber was set to continuously change the temperature from 27 to 40 °C, and the temperature change time was 400 min. The laser microdisplacement sensor was used to carry out real-time tracking of the amplitude characteristics under the three conditions of uncontrolled, piezoelectric feedback amplitude control and harmonic component control, respectively. The results are shown in Figure 7.

The results show that the amplitude shift under uncontrolled condition was about 3.344 μm and the amplitude change rate was 1.71%/°C. Under the piezoelectric feedback amplitude control condition, the amplitude shift was 0.749 μm and the rate of change was 0.38%/°C. The amplitude shift based on the harmonic component control was 0.211 μm and the rate of change was 0.11%/°C. In the temperature range of 27–40 °C, the amplitude stability was improved about 16 times by the amplitude control method based on the harmonic component of the modulated signal. Moreover, the proposed method can effectively reduce the amplitude drift caused by temperature change and the control stability was about 4 times higher than that of the piezoelectric feedback amplitude control method. The experimental results show that the control method based on the harmonic component of the modulated signal could significantly improve the stability of the modulation structure under the condition of variable temperature and its ability to suppress the amplitude temperature drift was also verified.

## 4. Conclusions

The effect of magnetic flux vertical modulation based on piezoelectric resonance was closely related to the amplitude stability of the modulation structure. It is found that the harmonic component of the modulated signal can reflect the amplitude characteristics. On this basis, a modulated structure amplitude control method based on the ratio of the first and second order harmonic components of the modulated signal is proposed in this paper. The test results show that the proposed control method can improve the amplitude stability by about 18 times under constant temperature. When the temperature is continuously increased from 27 to 40 °C, the proposed control method can improve the amplitude stability by about 16 times and the amplitude stability is about 4 times better than that of the piezoelectric feedback control method. 

The proposed control method uses the harmonic component of the modulated signal to establish the amplitude control loop, which can closely combine the sensor output signal with the structural characteristics. The control loop can be built directly on the original circuit system without the need of an independent amplitude conversion module. Experimental results show that this control method could greatly improve the amplitude stability and had significant advantages in temperature drift suppression. Its significance lies in that it cannot only improve the stability of the modulation structure itself, but also help to promote the adaptability of the TMR magnetic sensor to the complex variable temperature environment. More importantly, this control method greatly reduces the complexity of the system and the difficulty of process preparation. It provides a new idea for the optimization design of MEMS resonant control system. It is worth noting that the current control method still has room for improvement in robustness and resilience. By consulting relevant literature [14,15,16], we will carry out research on the robust control and resilient control of the magnetic flux modulation structure, which is of great significance for improving the reliability of the TMR magnetic sensor system in practical application.

## Data Availability

Experimental data can be obtained by contacting the author via email (zhangjunshengNUDT@126.com).
